# Comparison of Peritoneal Cytology Results Before and After Resection in Gastric Cancer Patients

**DOI:** 10.7759/cureus.65832

**Published:** 2024-07-31

**Authors:** Doruk Hacıoğlu, Erkan Guler, Tufan Gümüş, Sinan Ersin, Özgür Fırat, Taylan Özgür Sezer

**Affiliations:** 1 General Surgery, Ege University Medical Faculty, Deptartment of Surgery, Izmir, TUR; 2 General Surgery, Mersin University, Faculty of Medicine, Mersin, TUR

**Keywords:** survival, peritoneal cytology, metastasis, gastric cancer, cancer prognosis

## Abstract

Objective: Peritoneal cytology is used to detect the peritoneal spread of gastric cancer and to assess survival rate. The aim of this study was to compare the risk factors, recurrence, and survival of gastric cancer patients with positive and negative peritoneal cytology before and after resection.

Materials and methods: Patients with gastric cancer who underwent elective surgery were retrospectively analysed. The study covered a period between September 2018 and September 2020. After applying the exclusion criteria, 57 patients were included in the study. For the purpose of this study, peritoneal cytology was taken from the same three intra-abdominal regions before and after resection from patients with operable gastric cancer.

Results: Of the 57 patients included in the study, 36 (63.2%) were male patients and 21 (36.8%) were female patients. Preoperative or postoperative malignant cytology was detected in 12 patients (21.1%). Tumour diameter was larger in patients with preoperatively detected malignant cytology than in the patients with postoperatively positive malignant cytology (66.67 mm vs. 44.44 mm) (p = 0.006). The recurrence rate was higher in patients with preoperative and postoperative positive cytology than in those with negative cytology (p = 0.019). The survival of patients with preoperative malignant cytology was worse than the survival of patients with preoperative benign cytology (p = 0.011). A significant correlation was found between lymphovascular invasion (+), perineural invasion (+), T4, Stage 3 disease, number of malignant lymph nodes, and preoperative cytology positivity (p <0.05).

Conclusion: In our study, we found that the preoperative cytology positivity is associated with lymphovascular invasion positivity, perineural invasion positivity, T4 tumour, Stage 3 disease, and the number of malignant lymph nodes. Postoperative positive cytology was not associated with the same variables. Because of the significant associations in preoperative positivity, fluid samples should be obtained immediately after the abdomen is open and before the tumour is manipulated. If possible, fluid samples should be taken from different quadrants, but if the sample is to be taken from a single quadrant, it should be taken from the pelvis.

## Introduction

Gastric cancer remains the fifth most common cancer worldwide and the third leading cause of cancer-related deaths [1]. In 2020, 1,089,103 gastric cancer cases were diagnosed worldwide, and there were 768,793 deaths due to gastric cancer [2]. The definitive treatment of non-metastatic gastric cancer is surgical resection and perioperative chemotherapy, or chemoradiotherapy. However, the five-year survival is less than 5% for gastric cancer with metastatic or peritoneal spread [1-2].

At the time of diagnosis, it is challenging to determine whether the disease has spread to the peritoneum or to predict in which patient the disease might spread in the future. Peritoneal cytology sampling is widely used in the world during staging laparoscopy or definitive surgery to detect perioperative peritoneal spread and to evaluate survival rate. For patients whose peritoneal cytology resulted in malignancy by applying the conventional method, median survival time is measured in months, even with the macroscopic curative surgical resection [3]. Even though there is considerable information and assumptions about the peritoneal spread and cytology positivity, a consensus algorithm has not been reached worldwide. Furthermore, there is no definite consensus regarding the stage of treatment in patients with positive peritoneal lavage cytology. After opening the abdomen, we obtained the lavage cytology separately from three quadrants: the left upper quadrant, the right upper quadrant, and the pelvis, respectively. We also repeated this procedure in the same order just before the operation was completed and the abdomen was closed. One of the primary objectives of this study is to determine the microscopic peritoneal spread of patients without perioperative metastasis, evaluate their prognosis, and examine the relationship between conventional cytology positivity and other independent variables. Another goal is to make a possible contribution to the literature by comparing preoperative peritoneal cytology and postoperative peritoneal cytology.

## Materials and methods

This single-centre, retrospective study was conducted at the Department of General Surgery, Ege University Faculty of Medicine, Izmir, Turkey, between September 2018 and September 2020. The study included 116 patients older than 18 years who underwent elective open abdominal surgery due to gastric cancer. Patients who underwent total gastrectomy or distal gastrectomy + D2 lymph node dissection and whose intra-abdominal washing cytology were taken from the three quadrants were included. The study included patients who were diagnosed with gastric cancer with the pathology report of the endoscopic biopsy, who were shown to have no distant organ metastasis by radiological imaging (CT, MR, PET/CT), and who did not have a direct invasion of the tumour outside the stomach (T4). Patients who had ascites in the abdomen during laparotomy, where the tumour invaded other organs by exceeding the serosa and there were signs of peritoneal carcinomatosis and metastases in solid organs, were excluded from the study. Cytology samples were obtained from 72 of 116 patients operated on for gastric cancer. After exclusion criteria, 57 patients were included in the study. The study protocol was approved by the Ege University Faculty of Medicine ethics committee (approval number: 07.01.2021-E. 21-1T/17). The study was conducted in accordance with the principles of the Declaration of Helsinki.

After laparotomy for definitive surgery, 50 ml of isotonic sodium chloride (NaCl) solution was administered to the patients intra-abdominally in the next order: left upper quadrant/subphrenic region and right upper quadrant-/subphrenic region and pelvis, before any organ was manually manipulated and then aspirated. Thus, three abdominal washing fluids were obtained and marked as a “preoperative left upper quadrant," “preoperative right upper quadrant,” and “preoperative pelvis." After resection, anastomoses and hemostasis were achieved, and three more abdominal washing fluids were obtained in the same orders as preoperatively and marked as “postoperative left upper quadrant, postoperative right upper quadrant,” and “postoperative pelvis” just before the abdomen was closed. In total, six abdominal washing fluids from the three intra-abdominal quadrants for each patient were sent to pathology for cytological examination. At the pathology lab, the liquid samples were centrifuged at 2800 rpm for five minutes. The obtained material was spread on slides and fixed with 96% ethanol or methyl alcohol. Papanicolau and hematoxylin-eosin staining were performed and examined by an experienced cytopathologist.

Statistical analysis was performed using the IBM SPSS version 25.0 software (IBM Corp., Armonk, NY). Descriptive data were expressed in mean ± standard (SD), median (minimum-maximum), or number and frequency. Demographic data, surgery data, pathology results, recurrence, and survival rates were evaluated statistically. Patients with positive cytology in at least one of six cytological samples were accepted as patients with malignant cytology. Survival analysis and recurrent disease analysis were performed with the Kaplan-Meier test, and groups were compared with the log-rank test. The receiver operating characteristic (ROC) curve test was used to compare the tumour diameters of patients with malignant cytology and patients with benign cytology and to calculate the threshold value, sensitivity, and specificity in the relationship between tumour size and cytology positivity. A p-value of <0.05 was considered statistically significant.

## Results

**Figure 1:**
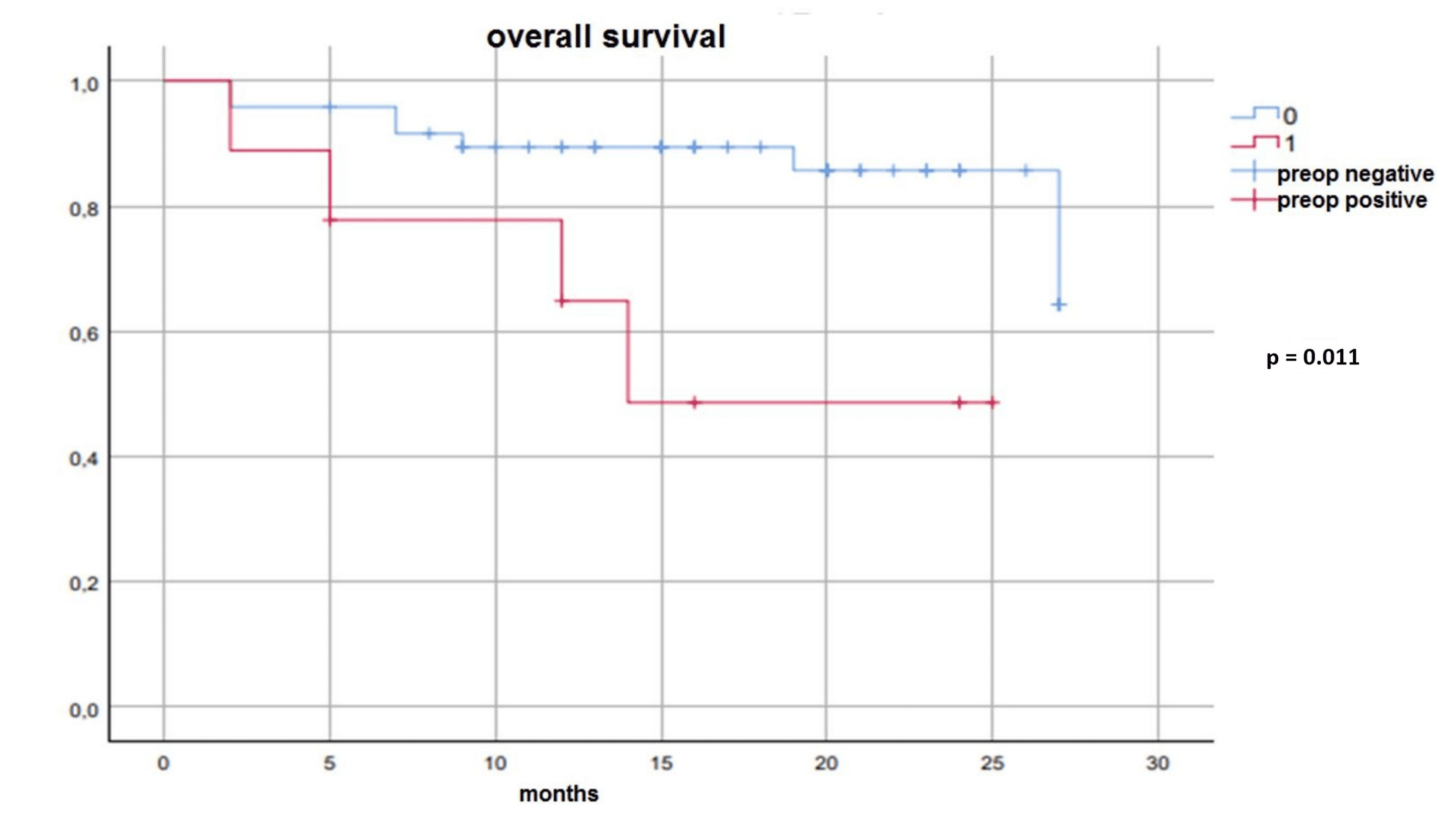
Survival analysis of preoperative (preop) patients with malignant cytology and patients with preoperative benign cytology

Fifty-seven patients were included in the study. Thirty-six patients were male (63.2%), and 21 were female patients (36.8%). The mean age of the patients was 62.12 (±11.54) (Table 1). Preoperative or postoperative malignant cytology was detected in 12 of 57 patients (21.1%), and this group was marked as 'patients with malignant cytology.' Although the number of excised lymph nodes in patients with malignant cytology was lower, the number of malignant lymph nodes was higher (p = 0.305). The relationship between T (tumour) and N (node) scores and cytology positivity was insignificant (Table 1). In this study, we categorized the patients into two groups based on their disease stage: 'Stage 3' and 'Others' (Stages 0, 1, and 2), with 37 patients in Stage 3 (64.9%) and 20 in the other stages (Stages 0, 1, and 2, 35.1%). Although not statistically significant, patients with malignant cytology were in a more advanced stage (p = 0.132). Tumour diameter was larger in patients with malignant cytology (p = 0.006) (Tables 1-2). The threshold value for cytology positivity was 43.5 mm. In tumours larger than 43.5 mm, cytology was positive, with 91.7% sensitivity and 53.3% specificity (Figure 1). Pelvic positivity was detected in eight out of 12 patients with cytology positivity. In four patients, positivity was detected in the left upper quadrant.

**Table 1:** Clinicopathological comparison of patients with positive cytology (preop or postop) and negative cytology TNM: tumour, node, metastasis

	Total	Positive cytology	Negative cytology	p-value
Number	57	12	45	
Age (years)	62.1(±11,5)	61.6(±10.8)	62.2(±11.9)	0.85
Sex				0.66
Male	36	7	29	
Female	21	5	16	
Surgical procedure				0,673
Total gastrectomy	35	8	27	
Distal gastrectomy	22	4	18	
Depth of tumor invasion				0,081
T0-1-2-3	27	5	22	
T4	30	7	23	
Lymph node metastasis (average)				0.35
Malign node/total	7.1/26.4	10/22	6.4/27.6	
TNM staging				0.132
Stages 0, 1, 2	20	2	18	
Stage 3	37	10	27	
Perineural invasion				0.276
Yes	35	9	26	
No	22	3	19	
Lymphovascular invasion				0,177
Yes	33	9	24	
No	24	3	21	
Histopathological types				0,115
Tubular	28	6	22	
Papillary	5	2	3	
Other type	24	4	20	
Tumor size (mm) average	49,1	66,6	44,4	0,006
Tumor localization				0,328
Cardia	13	3	10	
Corpus	22	5	17	
Antrum and pylorus	22	5	17	
Recurrence				0,019
No	12	5	7	
Yes	45	7	38	
Eksitus				0.085
Yes	11	4	7	
No	46	8	38	

**Table 2:** Clinicopathological comparison of patients with preoperative positive cytology and negative cytology TNM: tumour, node, metastasis

	Total	Positive cytology	Negative cytology	p-value
Number	57	9	48	
Age (years)	62.1(±11,5)	61.2(±12.8)	62.6(±11.4)	0.84
Sex				0.72
Male	36	5	31	
Female	21	4	17	
Surgical procedure				0,272
Total gastrectomy	35	7	28	
Distal gastrectomy	22	2	20	
Depth of tumor invasion				0,002
T0-1-2-3	27	0	27	
T4	30	9	21	
Lymph node metastasis = N score				0.120
N0	16	0	16	
N1	8	1	7	
N2	10	2	8	
N3	23	6	17	
TNM staging				0.016
Stages 0, 1, 2	20	0	20	
Stage 3	37	9	28	
Perineural invasion				0,009
Yes	35	9	26	
No	22	0	22	
Lymphovascular invasion				0,007
Yes	33	9	24	
No	24	0	24	
Histopathological types				0,099
Tubular	32	3	29	
Other type	25	6	19	
Tumor size (mm) average	49,1	68,8	45,4	0,015
Tumor localization				0,780
Cardia	10	2	8	
Corpus	25	3	22	
Antrum and pylorus	22	4	18	
Recurrence				0,010
Yes	12	4	8	
No	45	5	40	
Eksitus				0.011
Yes	11	4	7	
No	46	5	41	

**Figure 2:**
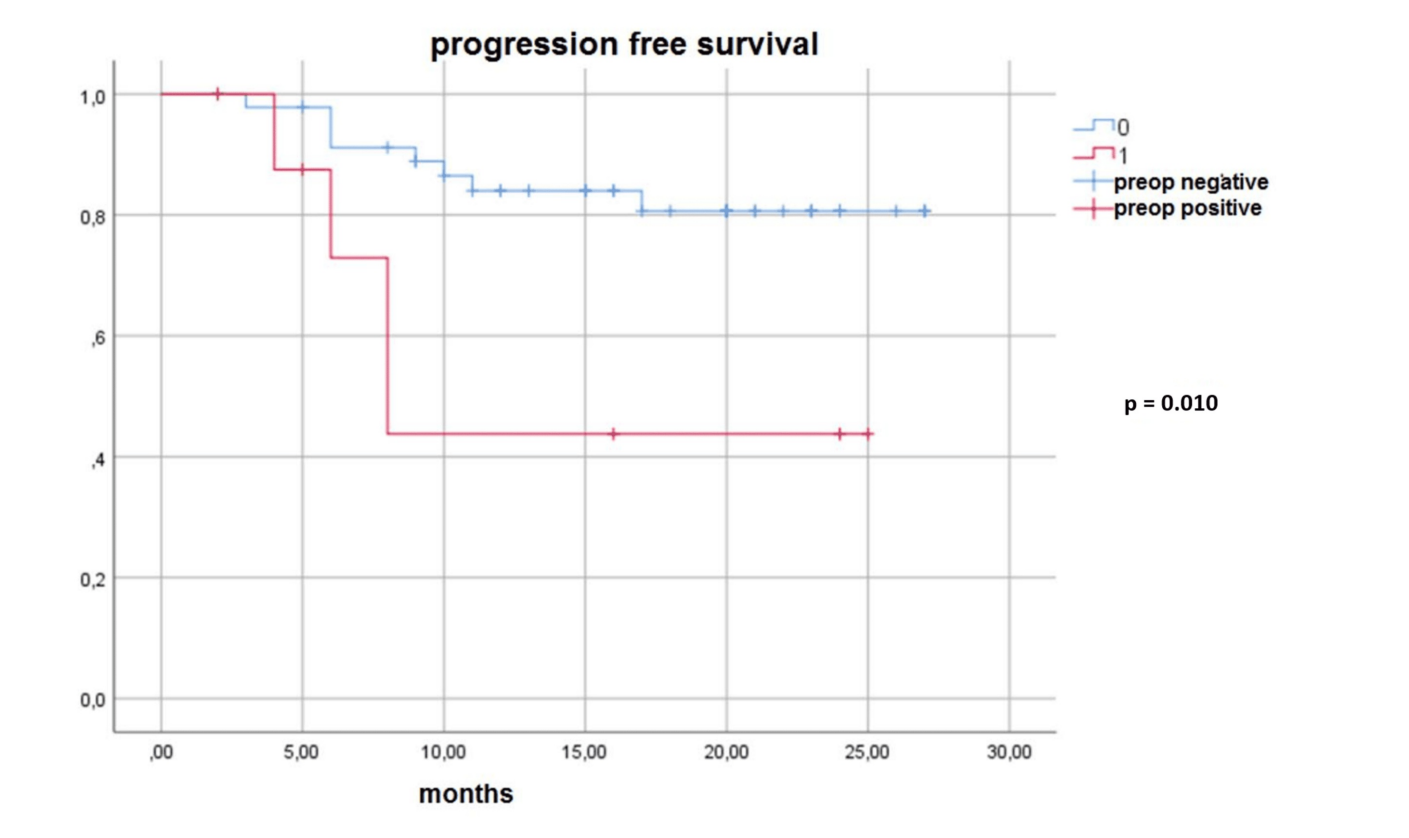
Survival analysis of preoperative (preop) patients with malignant cytology and patients with preoperative benign cytology

**Figure 3:**
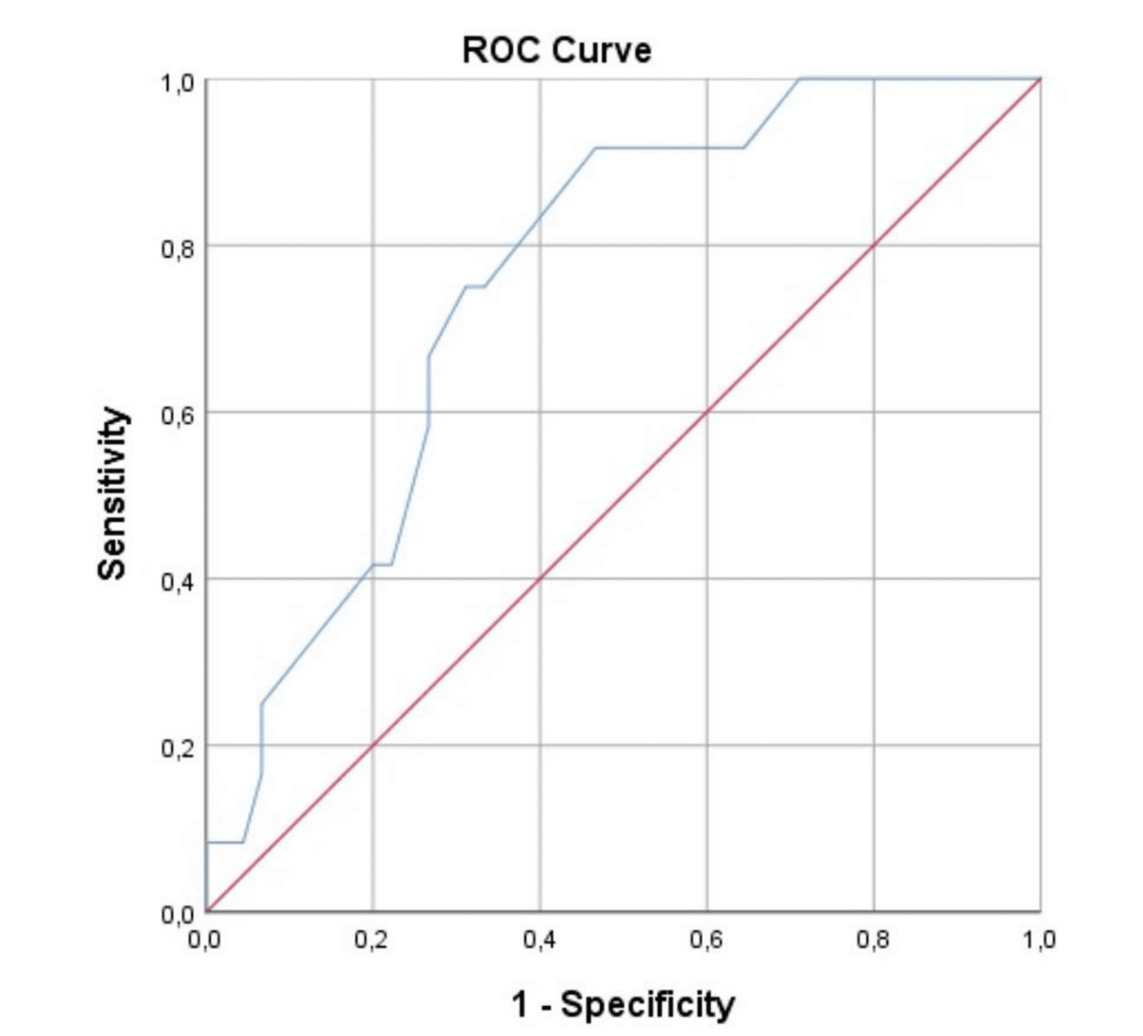
Receiver operating characteristic (ROC) curve of the tumour size in patients with positive cytology (preoperatively or postoperatively)

The recurrence appeared in five out of 12 patients with malignant cytology. The recurrence appeared in seven out of 45 patients with benign cytology. Recurrence was higher in patients with malignant cytology (p = 0.019). Kaplan-Meier survival analysis was used for patients with malignant cytology and patient groups with benign cytology. Seven of 45 patients with benign cytology and four of 12 patients with malignant cytology died. Malignant cytology positivity did not significantly affect the overall survival rate (p = 0.085). Four of nine patients with positive preoperative cytology died during the follow-up. Seven of 48 patients with negative preoperative cytology died during the follow-up. (Figures 2-3). Patients with preoperative benign cytology had better survival than patients with preoperative malignant cytology (p = 0.011). Postoperative cytology positivity did not affect survival (p = 0.163).

Preoperative cytology-positive patients and preoperative cytology-negative patients were compared in terms of recurrent disease. While four of nine preoperative cytology-positive patients had recurrent disease, eight of 48 preoperative cytology-negative patients had recurrent disease. More and faster recurrence or metastatic disease development was statistically significant in patients with preoperative malignant cytology (p = 0.010). Three of five patients with positive postoperative cytology had recurrent disease. Nine of 52 patients with negative postoperative cytology had recurrent disease. A higher number of recurrences or metastatic disease development and a shorter disease-free period were statistically significant in patients with postoperative malignant cytology (p = 0.021).

All nine preoperative cytology-positive patients had perineural invasion and lymphovascular invasion. There was a significant correlation between preoperative cytology positivity and perineural invasion and lymphovascular invasion positivity (p <0.05). There was no significant correlation between the T4 tumour and preoperative cytology positivity (p = 0.002). All preoperative cytology-positive patients were Stage 3. The patients were divided into two groups, “Stage 3” and “Others (stages 0, 1, and 2)." Chi-square analysis was performed, and there was no significant relationship between preoperative cytology positivity and Stage 3 disease (p = 0.016). There were an average of 11.03 malignant lymph nodes in patients with preoperative malignant cytology and an average of 6.17 malignant lymph nodes in patients with preoperative benign cytology. The Mann-Whitney test analyzed the relationship between excised lymph node counts, malignant lymph node counts, and preoperative cytology positivity. While there was no significant relationship between the number of excised lymph nodes and preoperative cytology positivity (p = 0.470), there was a significant relationship between the number of excised malignant lymph nodes and preoperative cytology positivity (p = 0.041).

## Discussion

The main findings of the current study are the factors affecting preoperative cytology positivity, and they include lymphovascular invasion positivity, perineural invasion positivity, T4 tumour, Stage 3 of the disease, and the number of malignant lymph nodes. In addition, preoperative cytology positivity is associated with recurrence and poor survival. Even in the absence of visible peritoneal implants, positive peritoneal cytology is considered M1 disease. The optional modalities have not been established for these patients.

The incidence of positive peritoneal cytology for patients with gastric cancer ranges from 4% to 41% [3-4]. In Japanese patients, this rate is about 5%-20% [5-6]. This study detected preoperative or postoperative malignant cytology in 12 (21.1%) of 57 patients. In a study of 371 patients, positive peritoneal cytology was noted to increase with the T stage (T1/T2: 2%, T3/T4: 10%, P = 0.02). However, in the same study, they didn't report a correlation between lymph node involvement, tumour location, and peritoneal cytology positivity [3].

There was a statistically significant correlation between T4 tumour and preoperative cytology positivity in our study but no association between general cytology positivity or postoperative cytology positivity. There was a significant correlation between preoperative cytology positivity and advanced disease (Stage 3). Tumor diameter was significantly larger in patients with malignant cytology. The threshold value for cytology positivity in tumour size was 43.5 mm. Cytology positivity was higher in tumours larger than 43.5 mm, with 91.7% sensitivity and 53.3% specificity. Consistent with the literature, the larger tumour had a more advanced stage and metastatic.

Recurrent disease was statistically significantly higher in patients with malignant cytology than in patients with benign cytology. A systematic review stated that cytology positivity in patients with gastric cancer was associated with the development of future recurrent disease and decreased mean survival [7]. In the absence of visible peritoneal metastases, positive peritoneal cytology has been associated with early recurrence and poor prognosis in many studies [3, 8]. In a 2018 meta-analysis, peritoneal cytology positivity was associated with shorter survival. However, peritoneal cytology positivity has better survival than macroscopic peritoneal spread [9]. Our study showed that patients with preoperative benign cytology had better survival rates than patients with preoperative malignant cytology (p = 0.011). There was also a significant correlation between preoperative cytology positivity, lymphovascular invasion positivity, and perineural invasion positivity (p = 0.007, p = 0.009). There was a significant correlation between the number of excised malignant lymph nodes and preoperative cytology positivity (p = 0.02). The same relationship was not shown in general cytology positivity and postoperative cytology positivity. With all these findings, we can say that preoperative cytology positivity is more valuable than postoperative cytology positivity and general cytology positivity.

The relationship between gender, age, type of operation, tumour localization, and lymph node metastasis with cytology positivity was not statistically significant. Except for the amount of lymph nodes, this situation is compatible with the literature. There is a clear difference between the groups in lymph node metastasis, but it is not statistically significant. According to a review, risk factors for cytology positivity in gastric cancer patients undergoing R0 resection differ in extensive studies. When the results of these studies are evaluated together, the common risk factors are increased T stage (having a T3-T4 tumour) and lymph node metastasis (>N1) [8]. However, it is seen that some of the studies included in the review have different risk factors. Some of those are increased stage, tumour size, serosal invasion, and serum carcinoembryonic antigen (CEA) levels [8]. In general, our findings were consistent with the literature.

The success of conventional cytology in showing free tumour cells in the intra-abdominal cavity is controversial. Many biomarkers in peritoneal fluid have been studied as an alternative to traditional cytology. Examination of CEA mRNA expression by CEA-specific reverse transcription-polymerase chain reaction (RT-PCR) analysis is the most emphasized among these methods. The RT-PCR technique is more sensitive than conventional cytological examination in detecting cancer cells and predicting peritoneal recurrence [10]. The immunohistochemical examination result of intraoperative peritoneal washing fluid shows free cancer cells in the peritoneum compared to conventional cytology; the T score, N score, and its relationship with lymphovascular invasion and the survival effect are more clear [11]. Although many researchers have shown that molecular techniques using RT-PCR can serve as a helpful method for detecting free cancer cells, there are still a few problems. The RT-PCR test is time-consuming, expensive, and relatively laborious compared to conventional cytology; accuracy varies greatly between laboratories; and methods for processing peritoneal washes are not yet standardized. There are still many limitations that must be overcome before a genetic diagnosis can be accepted as a routine experiment to detect free intraperitoneal cancer cells. For a reliable method to see free cancer cells, the process and practice of genetic detection by peritoneal washes must be standardized, and simple diagnostic devices and readily available kits must be developed [12].

In a study, the significant causes of postoperative cytology positivity in gastric cancer were cancer cells shed from the opened gastric lumen and cancer cells shed from the cut lymphovascular pedicles [13]. In another study, the rate of conversion of cytology that was negative preoperatively to positive postoperatively was 12%, and the possible reasons for this were based on similar reasons [14].

The conversion of preoperative negative cytology to postoperative positive cytology in our study may be due to the following reasons: shedding of cancer cells from the serosa; shedding of cancer cells from the capillary vessels and lymphatic pathways clogged by the tumour cut during the operation; cancer cells shed from the gastric lumen, which was unintentionally opened during the operation; contaminated transport of cancer cells to the peritoneum by an operating instrument; and preoperative cytology false negative.

On the other hand, seven patients were positive preoperatively and negative postoperatively. These data show that targeted oncologic surgery is generally performed successfully.

In another study, peritoneal cytology from the pelvis was negative in 17% of patients while positive in other quadrants. This study found the highest positivity rate in the right subhepatic region with 83%, followed by 74.8% in the left subphrenic area [15]. In our study, eight of 12 patients with cytology positivity had positive samples from the pelvis (66%). Left upper quadrant cytology was positive in four patients (33%). In light of these data, although more studies are needed for the best first- and second-site alternatives, we think that samples taken from multiple cavities increase the sensitivity of peritoneal cytology and are effective in predicting the peritoneal spread of cancer cells and the prognosis of the patient.

However, there are also some limitations to this. First, this study has a single-centre, retrospective, non-randomized design with a relatively small sample size. This can be attributed to the necessarily dramatic decrease in the number of surgeries as of February 2020 due to the COVID-19 pandemic. The mean follow-up period was 16.3 months. However, 12 out of 57 patients had recurrent disease, and 11 died in a short follow-up period. Noteworthy, 64% of the patients were Stage 3, so it is expected to see so many recurrences and mortality in such a short time. In addition, the literature was consistent with the survival-recurrence rates. Performing the study with conventional cytology decreased the sensitivity. In previous studies, the sensitivity of traditional peritoneal cytology to show the extent of peritoneal spread was 64%-89% [16, 17]. The inclusion of newer and more sensitive cytological examinations (such as RT-PCR) will increase the power of the study. In gastric cancers without known metastasis and peritoneal carcinomatosis, patients with malignant intraoperative peritoneal washing cytology have been shown to have a reduced recurrence rate and improved survival with adjuvant chemotherapy [18]. Peritoneal carcinomatosis has been shown to develop in the future in 25% of patients with negative cytology who were evaluated as localized gastric carcinoma, who underwent resection, and who received adjuvant chemotherapy [19]. The adjuvant treatment status of the patients should be included in the data, and the survival and recurrent disease statistics should be updated in the following years.

## Conclusions

Preoperative cytology positivity is associated with perineural invasion positivity, T4 tumour, Stage 3 disease, and a high number of malignant lymph nodes. In addition, preoperative cytology positivity is associated with a higher incidence of recurrence and worse survival. Therefore, peritoneal washing cytology samples from patients operated on for gastric cancer and without known metastasis and peritoneal carcinomatosis should be obtained immediately after the abdomen is opened and before the tumour is manipulated. Cytology samples should be taken from multiple quadrants, if possible. If it is to be taken from a single localization, it will be appropriate to take it from the pelvis. There is a need for randomized controlled studies, if possible, involving a larger number of patients and examining the abdominal washing fluids with advanced molecular tests.
